# Antibacterial, Hydrophilic Effect and Mechanical Properties of Orthodontic Resin Coated with UV-Responsive Photocatalyst

**DOI:** 10.3390/ma11060889

**Published:** 2018-05-25

**Authors:** Akira Kuroiwa, Yoshiaki Nomura, Tsuyoshi Ochiai, Tomomi Sudo, Rie Nomoto, Tohru Hayakawa, Hiroyuki Kanzaki, Yoshiki Nakamura, Nobuhiro Hanada

**Affiliations:** 1 Department of Orthodontics, Tsurumi University School of Dental Medicine, Yokohama 230-8501, Japan; 2711005@stu.tsurumi-u.ac.jp (A.K.); sudo-tomomi@tsurumi-u.ac.jp (T.S.); kanzaki-h@tsurumi-u.ac.jp (H.K.); nakamura-ys@tsurumi-u.ac.jp (Y.N.); 2 Department of Translational Research, Tsurumi University School of Dental Medicine, Yokohama 230-8501, Japan; hanada-n@tsurumi-u.ac.jp; 3 Photocatalyst Group, Research and Development Department, Local Independent Administrative Agency Kanagawa Institute of industrial Science and TEChnology (KISTEC), 407 East Wing, Innovation Center Building, KSP, 3-2-1 Sakado, Takatsu-ku, Kawasaki, Kanagawa 213-0012, Japan; pg-ochiai@newkast.or.jp; 4 Materials Analysis Group, Kawasaki Technical Support Department, KISTEC, Ground Floor East Wing, Innovation Center Building, KSP, 3-2-1 Sakado, Takatsu-ku, Kawasaki, Kanagawa 213-0012, Japan; 5 Photocatalysis International Research Center, Tokyo University of Science, 2641 Yamazaki, Noda, Chiba 278-8510, Japan; 6 Department of Dental Engineering, Tsurumi University School of Dental Medicine, Yokohama 230-8501, Japan; nomoto-r@tsurumi-u.ac.jp (R.N.); hayakawa-t@tsurumi-u.ac.jp (T.H.)

**Keywords:** orthodontic resin, photocatalyst TiO_2_, antibacterial, cariogenic, early colonizer, hydrophilic properties, irradiation

## Abstract

Photocatalysts have multiple applications in air purifiers, paints, and self-cleaning coatings for medical devices such as catheters, as well as in the elimination of xenobiotics. In this study, a coating of a UV-responsive photocatalyst, titanium dioxide (TiO_2_), was applied to an orthodontic resin. The antibacterial activity on oral bacteria as well as hydrophilic properties and mechanical properties of the TiO_2_-coated resin were investigated. ultraviolet A (UVA) (352 nm) light was used as the light source. Antibacterial activity was examined with or without irradiation. Measurements of early colonizers and cariogenic bacterial count, i.e., colony forming units (CFU), were performed after irradiation for different time durations. Hydrophilic properties were evaluated by water contact angle measurements. While, for the assessment of mechanical properties, flexural strength was measured by the three-point bending test. In the coat(+)light(+) samples the CFU were markedly decreased compared to the control samples. Water contact angle of the coat(+)light(+) samples was decreased after irradiation. The flexural strength of the specimen irradiated for long time showed a higher value than the required standard value, indicating that the effect of irradiation was weak. We suggest that coating with the ultraviolet responsive photocatalyst TiO_2_ is useful for the development of orthodontic resin with antimicrobial properties.

## 1. Introduction

Maintenance of good oral hygiene after an active orthodontic treatment is one of the most important procedures. In general, after an active orthodontic treatment, moved teeth and jawbone are retained by acrylic resin based retainer [1]. The retainer is typically used for at least two years. The longer the retainer is used, the more unhygienic it becomes. Gradually, micro-organisms colonize on the surfaces of retainers, just as on dentures, and they often cause stomatitis, dental caries, periodontal disease, chronic atrophic or candidiasis [2,3,4,5,6,7,8,9,10,11]. Porosities on the outer and inner surfaces of retainer also provide favorable conditions for microbial colonization [12]. Therefore, prevention of micro-organism colonization is essential for the maintenance of good oral hygiene and prevention of oral diseases.

Management of oral biofilm is important to maintain a good oral status. Common oral diseases, dental caries and periodontitis, are caused by an imbalance between biofilms and host defenses [13]. The initial process in the oral biofilm formation starts with pellicle, covering the tooth surface within a few minutes after mechanical cleaning. The pellicle plays a major role in the development and maintenance of bacterial communities. Then, the complex bacterial communities develop on the pellicle within a few days, and their components can be divided into two categories—early colonizers, and late colonizers [14]. Early colonizers that directly adhere to the pellicles are predominantly streptococci [15]. Streptococci constitute 60% to 90% of the bacteria that colonize on the teeth in the first 4 h after professional cleaning [16]. These species are mainly Gram-positive and have minor pathogenic effects on periodontal tissue. Late colonizers, such as *Fusobacterium nucleatum*, *Porphyromonas gingivalis*, *Tannerella forsythia*, *Treponema denticola*, and *Aggregatibacter actinomycetemcomitans* tend to be more pathogenic than the early colonizers. The late colonizers alone cannot form a biofilm on the tooth surface, but they form a biofilm by their parasitical adherence to the early colonizers [14].

In the formation of a biofilm it is inevitable that colonizing bacteria primarily adhere to the surface of the retainer [17]. Therefore, it is critical to prevent the bacteria from adhering to the retainers. As the orthodontic acrylic resin and denture base acrylic resin have similar requirements for clinical use (ISO 20795), the results for the denture base resin are also applicable for orthodontic acrylic resin. Various approaches have been employed for the prevention of microbial biofilms. These include dental disinfection, denture cleaner, mixing resin with antibacterial agents, and coating resin with antiseptic [6,18,19,20,21,22,23,24,25,26,27,28,29,30,31,32]. However, the biofilms are resistant to antibacterial and antifungals agents [33,34]. Moreover, a long-term use of denture cleanser corrodes metals such as clasps [35,36]. Denture cleanser affects the color stability of the denture base acrylic resin [37]. Mechanical cleaning with the adjunctive use of antimicrobial solutions is helpful in reducing biofilm growth or preventing its formation. However, such approaches rely primarily on a patient’s compliance, and may be compromised in pediatric, geriatric, and handicapped individuals. Silver nanoparticles impregnated in acrylic resin make the appliance antibacterial, but releasing silver nanoparticles from the resin is a limiting factor [24,25,27,31]. Incorporating fluorine and silver ions into resin elutes antimicrobials but it is available only for the first few weeks [27]. Moreover, elution of antimicrobial agents may result in deterioration of the mechanical properties of the retainer over time. This reduction renders the appliance more susceptible to fracture, due to its low resistance to impact, low flexural strength, or low fatigue strength [38]. Hence, novel and alternative methods to prevent the micro-organism colonization are required.

Application of a photocatalyst is one of the effective and safe approaches to remove biofilm from the dentures or retainer [39,40]. Photocatalyst reaction is defined as a photocatalyst promoted reaction on a solid surface, usually a semiconductor [41]. Titanium dioxide (TiO_2_) is the most studied photocatalyst [39,41,42,43,44,45,46,47,48,49,50]. TiO_2_ is biocompatible, nontoxic, and inexpensive. TiO_2_ generates reactive oxygen species (ROSs) upon ultraviolet A (UVA) irradiation, and its strong oxidative power decomposes micro-organisms and organic materials [33,42,43,44,46,50]. Further, the photocatalyst reaction can obtain superhydrophilic properties by UV irradiation, and superhydrophilicity prevents dirt accumulation on the device.

The purpose of the present study was to test the clinical applications TiO_2_ coated orthodontic resin based retainer. To this end, we investigated the antibacterial effects as well as mechanical, and hydrophilic properties of acrylic based orthodontic resin coated with the photocatalyst TiO_2_ after irradiation with UVA. Thus, we determined its clinical suitability for use as an orthodontic retainer material. First, we examined the effect of the orthodontic resin coated with TiO_2_ on bacteria for various irradiation durations. Further, we investigated the antimicrobial effect against early colonizers, the bacteria which are first attached to the appliances. The effects on *S. mutans* and *S. sobrinus*, which are the most well-known cariogenic bacteria, were investigated. Second, mechanical properties of the orthodontic resin are evaluated by irradiating for about 2 years, which is the recommended usage period of the orthodontic retainer. Third, the hydrophilic properties were investigated as one of the photocatalytic effects. Acquiring hydrophilic properties by decreasing the contact angle could lead to the prevention of bacterial adhesion and a self-cleaning function.

## 2. Materials and Methods

Autopolymerizable orthodontic acrylic resin (Ortho Crystal, Nissin Co., Tokyo, Japan), which consisted of a liquid component and a powder component, was used for this study. In the following sections, autopolymerizable orthodontic acrylic resin are referred to as ‘resin’.

### 2.1. Sample Preparation

According to the manufacturer’s instructions, a powder-to-liquid ratio of 10 g:4.5 mL was used. Powder and liquid components were mixed under vibration for homogenization and removal of the trapped air. The slurry was poured into an aluminum open mold and pressed using a pair of glass plates to fabricate the specimens of different dimensions: 50 mm × 50 mm × 3.0 mm (*n* = 60), 50 mm × 50 mm × 3.0 mm (*n* = 20), and 64 mm × 10 mm × 3.5 mm (*n* = 35) for the antibacterial properties tests, photoinduced hydrophilic tests, and mechanical properties tests, respectively. The slurry resin was immediately transferred in the polymerization equipment for a dental technique at 40 °C (manufacturer’s recommendation: 30–40 °C), and 0.25 MPa for 30 min to enhance curing (Fit Resin Multicure, Shofu Inc., Kyoto, Japan). Following preparation, each specimen was kept at room temperature in water for 12 h to eliminate the residual monomer. All test specimens were gradually grinded with waterproof polishing paper, having a grain size of approximately 30 μm (P500), 18 μm (P1000), and 15 μm (P1200). The specimens were then divided into four test groups for the assessment of the antibacterial properties and water contact angle measurement of base resin coated with thin film of photocatalytic TiO_2_. Uncoated resin and non-lighted resin were used as control groups for their respective experimental group.

A spin-coating methods was used to apply ultraviolet-light-responsive photocatalytic titanium dioxide (UV-TiO_2_) to the surface of the materials. The surface modification of the specimen with commercialized photocatalytic TiO_2_ (NRC 350A and 360C, Nippon Soda Co., Ltd., Tokyo, Japan) was carried out by a sol-gel thin film spin-coating method according to the manufacturer’s instructions. NRC 350A was coated to the surface of the materials. After coating, they were dried in a desiccator under 30 °C for 48 h. Then NRC 360Cwas coated, and dried in a desiccator in the same way. After the coating, the surface conditions of the materials were observed by a scanning electron microscope (SEM; JSM-5600LV, JEOL, Tokyo, Japan) at an accelerating voltage of 15 kV. Specimens were sputter-coated with Au prior to the SEM observations.

We covered samples with a glass to prevent drying, and irradiation was carried out from above. UVA from a black light source (wavelength: 352 nm, FL15BLB, Toshiba Co., Tokyo, Japan) was selected as the light source for catalytic excitation. The irradiation was performed at a distance of 10 cm (1.0 mW/cm^2^ under the glass).

### 2.2. Bacterial Strains

*Streptococcus mutans* ATCC 25175 (*S. mutans*), *Streptococcus sobrinus* ATCC33478 (*S. sobrinus*), *Streptococcus gordonii* ATCC 10558 (*S. gordonii*), *Streptococcus oralis* ATCC 35037 (*S. oralis* ATCC), *Streptococcus oralis* GTC 276 (*S. oralis* GTC), *Streptococcus sanguinis* ATCC 10556 (*S. sanguinis*), and *Streptococcus mitis* MRS 08-31 (*S. mitis*) were used in this study. *S. mutans* and *S. sobrinus* are cariogenic bacteria. *S. gordonii*, *S. oralis* ATCC, *S. oralis* GTC, *S. sanguinis*, and *S. mitis* are the early colonizers. These bacterial species were inoculated into 4 mL of Tryptic Soy (TS) broth (Becton, Dickinson and Company, Sparks, MD, USA) and were cultured aerobically at 37 °C for 16 h. They were harvested by centrifugation at 3000× rpm for 15 min and then suspended with phosphate buffered saline (PBS) resulting in an optical density at 540 nm (OD_540_ equal to 1.0).

### 2.3. Antibacterial Test

A micro-organism suspension (500 µL) adjusted to OD_540_ = 1.0 was dropped directly on the surface of the coated and non-coated specimen on ice, and UV irradiation was performed for 0, 15, 30, 60, 90, 120, 150, and 180 min. After irradiation, each bacterial cell pellet was suspended in 1 mL PBS and subjected to serial 10-fold dilutions in PBS. The dilutions of each bacteria were inoculated on MS agar (Difico Mitis Salivarius Agar (semi-selective medium for streptococci); BD Biosciences, Flanklin Lakes, NJ, USA) in petri dishes with spiral plating equipment (Eddy Jet, IUL SA, Barcelona, Spain), and petri dishes were incubated under anaerobic conditions in an AnaeroPack-Anaero box (AnaeroPack System, Mitsubishi Gas Chemical Co., Inc., Tokyo, Japan) at 37 °C for 48 h. The number of colonies was counted in accordance with the spiral plater instruction manual. Each measurement was repeated three times.

### 2.4. Bending Test

The rectangular plates (64 mm × 10 mm × 3.5 mm) were immersed in distilled water at 37 °C for 0, 200, 400, 600, 800, 1000, 1200 h under UVA irradiation from a distance of 10 cm. The three-point bending test was conducted using a universal testing machine (EZ Test 500 N, Shimadzu Co., Kyoto, Japan) at a crosshead speed of 5 mm/min and a span length of 50.0 mm (*n* = 5). Then, load-displacement curves were plotted to measure bending strength, elastic modulus, and toughness. The test was conducted according to ISO20795-2 standard.

### 2.5. Water Contact Angle Measurement

The rectangular plates (50 mm × 50 mm × 3.0 mm) were prepared as indicated above and spin-coated with 125 μL of the experimental coating materials on each sample. The water contact angles were measured using a contact angle device (FTA125, First Ten Ångstroms, Portsmouth, VA, USA) at 25 °C. For surface analysis of the hydrophilic characteristics, 3.5 μL of deionized water (Milli-Q Plus system, Japan Millipore, Tokyo, Japan) was dropped on the surface, and video images were taken. Video images were automatically inputted to an attached computer in which the contact angles were measured using an image analysis program (FTA32 video, First Ten Ångstroms). Water contact angles were measured every 30 min for a period of 20 s at 25 °C.

### 2.6. Statistical Analysis

Antibacterial effects were analyzed by three factors: TiO_2_ coating, presence of UVA, and irradiated time (*n* = 3). The significance of differences in the antibacterial effects was examined using three-way ANOVA or two-way ANOVA. Mechanical properties were examined using one-way ANOVA, with irradiated time included as a factor (*n* = 5). Tukey’s HSD test was then used to determine the positions of significance. All statistical analyses were performed using IBM SPSS Statistics version 22.0 (IBM, Tokyo, Japan). A significance level of *p* < 0.05 was used.

## 3. Results

### Antibacterial Test

**Figure 1:**
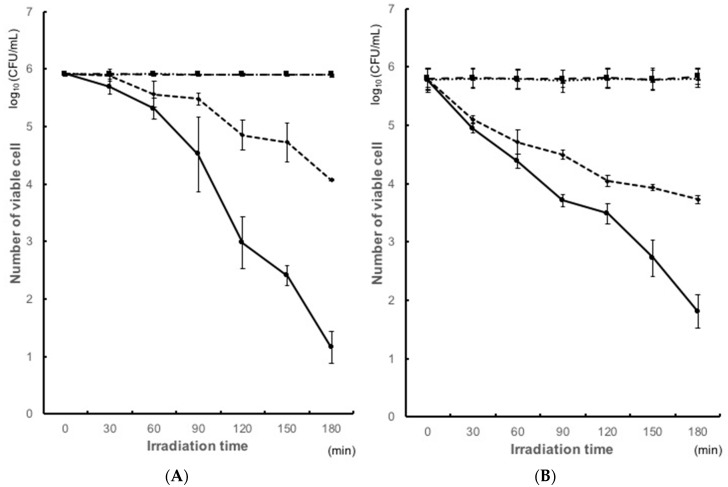
Antibacterial effects of TiO_2_ photocatalysis against (**A**) *Streptococcus gordonii*; (**B**) *Streptococcus oralis* ATCC. 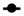 coat(+)light(+): experiment group containing powdered TiO_2_ with irradiation. 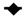 coat(−)light(−): control group without both TiO_2_ and irradiation. 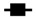 coat(+)light(−): experiment group in the presence of powdered TiO_2_ without irradiation. 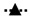 coat(−)light(−): experiment group without TiO_2_, but with irradiation.

The antimicrobial activity of the resin coated with TiO_2_ was examined by bacterial count of the early colonizers and cariogenic bacteria under UV irradiation. Figure 1 shows the antibacterial activity of the TiO_2_-coated resin surfaces against *S. gordonii* ATCC 10558, and *S. oralis* ATCC 35037 after irradiation. The coat(−)light(−) group was used as control group. The coat(+)light(−) group showed no significant differences compared with the control group. Hence, there was no effect with the coat alone. We also examined the activity with UV alone. Coat(−)light(+) induced a significant reduction in the number of colony. The number of *S. gordonii* was reduced from 1.6 × 10^7^ colony-forming units/mL (CFU/mL) to 1.6 × 10^6^ CFU/mL (after 120 min of UV irradiation), and that of *S. oralis* ATCC was reduced from 6.5 × 10^5^ CFU/mL to 3.1 × 10^4^ CFU/mL (after 90 min of UV irradiation). Consequently, about 90%, 95% colonies of *S. gordonii* and *S. oralis* ATCC were not formed on the coated plates upon irradiation (Figure 1A,B). On the other hand, coat(+)light(+) group showed a significant reduction in the CFU. The number of *S. gordonii* was reduced from 1.6 × 10^7^ CFU/mL to 2.7 × 10^4^ CFU/mL (after 120 min of UV irradiation) and that of *S. oralis* ATCC from 6.5 × 10^5^ CFU/mL to 5.2 × 10^3^ CFU/mL (after 90 min of UV irradiation). Thus, about 99.9% or more colonies of both *S. gordonii* and *S. oralis* ATCC were not formed on the coated plates upon irradiation (Figure 1A,B). Hence, coat(+)light(+) group showed a hundred times higher antibacterial effect, when compared with coat(−)light(+).

**Figure 2:**
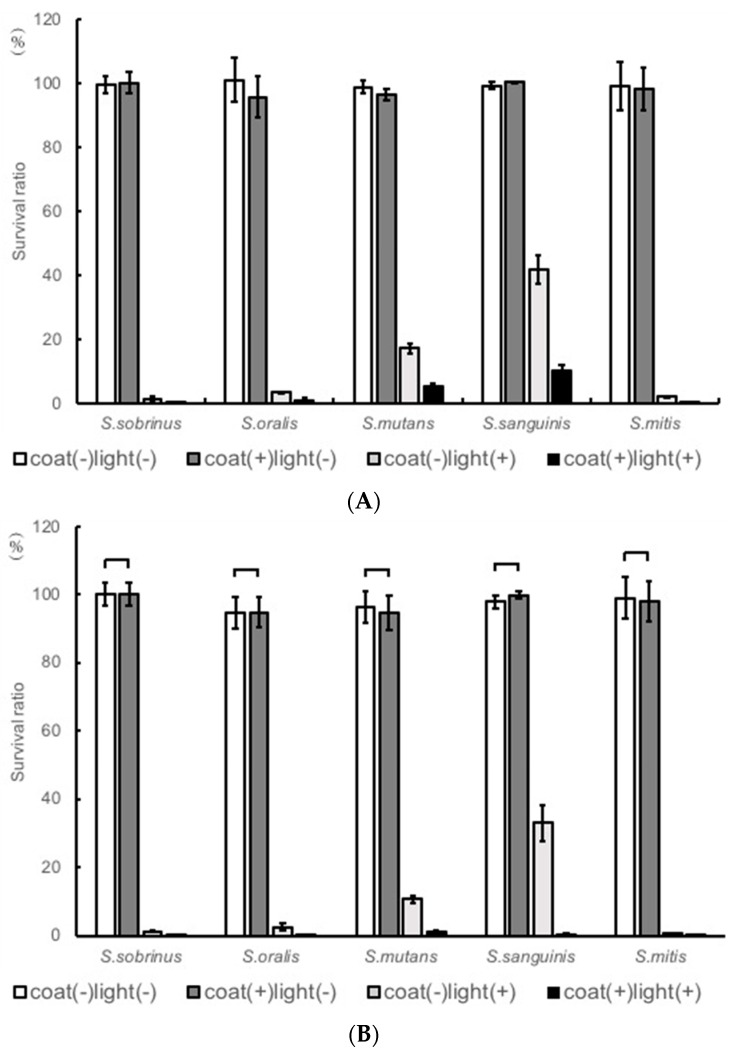
Antibacterial effects on various specimens of *Streptococcus sobrinus*, *Streptococcus oralis* GTC, *Streptococcus mutans*, and *Streptococcus sanguinis* at (**A**) 90 min, and (**B**) 120 min of ultraviolet A (UVA) irradiation.

Compared with the TiO_2_-noncoated samples, TiO_2_-coated samples showed a rapid decrease in the level of CFU of *Streptococcus gordonii*, particularly in the early stages (90 min and 120 min) of irradiation. Therefore, other strains were also examined at 90 and 120 min (Figure 2A,B). The cell viability on each sample before UV irradiation was set as 100%. The coat(+)light(+) clearly showed a great reduction in the cell viability. Cell viability was reduced to 0.2% for *S. sobrinus*, 0.9% for *S. oralis* GTC, 5.4% for *S. mutans*, and 9.9% for *S. sanguinis*. Clearly, the coat(+)light(+) samples exhibited the best antimicrobial performance in all microbes after 90 min UV irradiation. Similar results were observed even after 120 min of irradiation.

Table 1 shows explanatory variables related to bacterial counts by three way ANOVA. The coefficients of bacterial counts in light(+) and interaction of light(+) and coat(+) were all negative. The coefficients of light(+) for *S. oralis* and *S. sobrinus* were higher than those by interaction of light(+) and coat(+). In contrast, the coefficients of light(+) of *S. gordonii*, *S. mutans*, and *S. sanguinis* were higher than those by interaction of light(+) and coat(+). The coefficient of both light(+) and interaction of light(+) and coat(+) of *S. mutans* was the highest in all the bacteria. The coefficient for irradiation time of 120 min were significantly different in all bacterial species. In all the bacteria, the coefficient of coat(+) was almost 0. On the other hand, the coefficient of light(+) and interaction of light(+) and coat(+) were statistically significant. These results indicated that the antibacterial effect of the photocatalyst was exerted by UVA irradiation. It also showed differences in susceptibility of oral bacteria to UVA.

**Figure 3:**
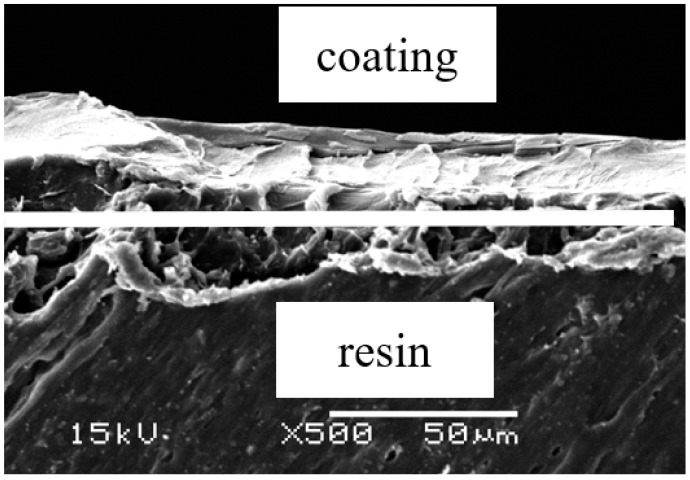
Scanning electron microscopy of cross sectional photographs of the TiO_2_ coating.

SEM was used to observe the cross section of coating; cross sectional photographs are shown in Figure 3.

**Figure 4:**
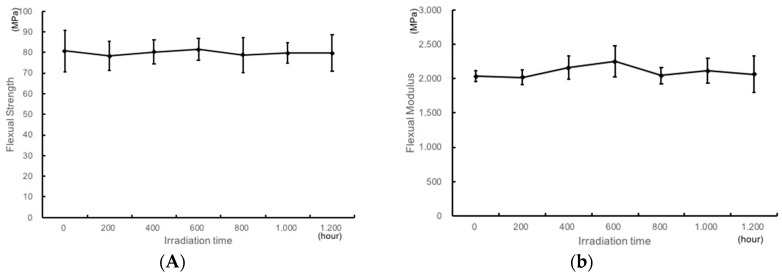
Flexural strength (**A**) and Flexural modulus (**B**) of the TiO_2_-coated resin plates upon UV irradiation.

The Flexural strength (Fs) and flexural modulus (Fm) of resin plates after UV irradiation are shown in Figure 4A,B, respectively. There was no difference between the irradiated test pieces, and all irradiated specimens fulfilled the requirements of the ISO 20795-2:2010 standard for Fs testing after 1200 h of UV irradiation (>65 MPa) (Figure 4A).

In the same way, all irradiated TiO_2_-coated specimens fulfilled the requirements of the ISO 20795-2:2010 standard for Fm testing after 1200 h of UV irradiation (>2000 MPa) (Figure 4B).

**Figure 5:**
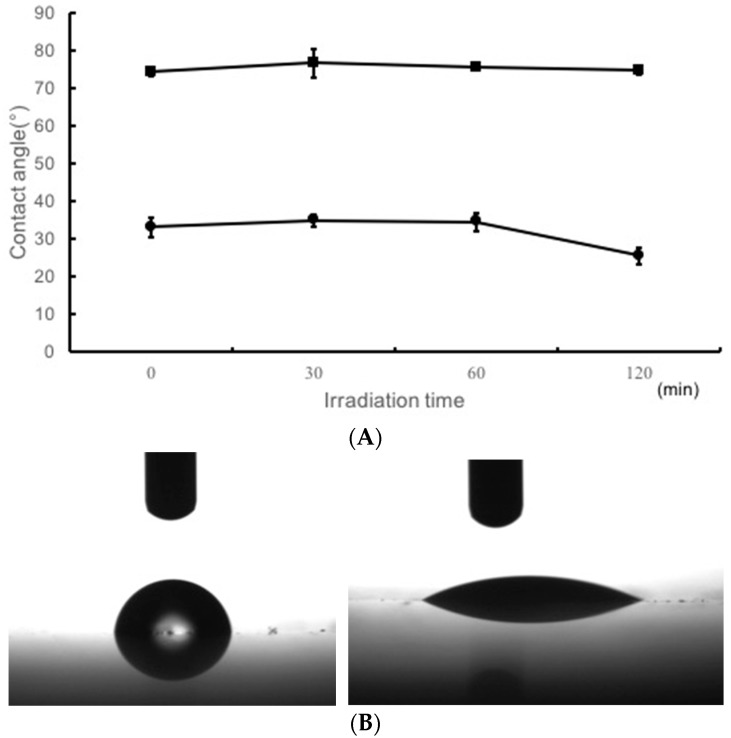
(**A**) Water contact angle of the resin plates with TiO_2_-coating upon UV irradiation for 0, 30, 60, and 120 min. (**B**) Image shows contact angle of a water droplet on non-coated (**left**) and TiO_2_-coated resin plate (**right**) after 120 min of UV irradiation.

Figure 5A shows the water contact angle for TiO_2_-noncoated groups and TiO_2_-coated groups. Figure 5B shows the images illustrating the wettability of water on TiO_2_-coated specimens or TiO_2_-noncoated specimens after 120 min of UV irradiation.

The water contact angle of TiO_2_-coated specimen was very small from the start of experiment, compared to that of TiO_2_-noncoated and it gradually decreased with time.

## 4. Discussion

This study demonstrates the antibacterial effects of TiO_2_-coating on *S. mutans*, *S. sobrinus*, and early colonizers upon UVA irradiation. When TiO_2_-coating and UVA were used together, a significant reduction in the microbial count was observed. In a previous study, TiO_2_ coated orthodontic arch wires showed the photocatalytic antibacterial effects on *S. mutans*, and its reduction rate was more than 99.99% by bacterial count after 1 h irradiation [51]. Also, a tissue conditioner containing a TiO_2_ photocatalyst decreased bacterial counts for *Escherichia coli* (about 90%), *Staphylococcus aureus* (>99.99%), and *S. mutans* (about 90%) after 2 h of irradiation [52]. Moreover, the photocatalytic antibacterial effects of metal specimens coated with two crystalline forms of TiO_2_ by thermal and anodic oxidation decreased bacterial counts for *S. mutans* (about 90%) after 60 min irradiation [43]. Furthermore, titanium disks coated with anatase-rich titanium dioxide (TiO_2_) reduced amount of viable cells of *S. oralis* by 40% after 24 h UVA exposure [53]. Interestingly, anodized titanium (AO) decreased survival ratio of *S. sanguinis* (70%) upon 2 h of UV irradiation [50]. Also, after 20 min of UV exposure to TiO_2_ surfaces, viabilities of *S. mutans* were reduced by 65% [54]. There were a few reports on *S. gordonii* and *S. sobrinus*. Finally, our results showed the bacterial count reduction rate of *S. gordonii* (>90%) (Figure 1), *S. mutans* (87%), *S. sobrinus* (>99%), *S. oralis* (98%), *S. sanguinis* (90%), and *S. mitis* (>99%) (Figure 2) after 90 min irradiation. The reduction rates of *S. oralis*, and *S. sanguinis* were better than those of past study [50,53]. The reduction rate of *S. mutans* was similar as previous studies [43,52,53,54]. However, a greater reduction rates of bacteria than those in our study were reported [51]. Although factors that contribute to the difference in the reduction rate are unknown, this may be due to the difference in the components of the photocatalyst used. This may also be due to the difference in the surface properties of the coating. Similar to the previous reports, the photocatalytic reaction induced a relatively mild decrease in the bacterial counts after the first 20 min of irradiation and showed a rapid decrease upon subsequent irradiation (Figure 1A) [55]. There is a difference in reaction by bacteria, but only upon irradiation for at least 90 min. Also, compared to conventional cleaning methods, cleaning is facilitated by coating TiO_2_. This may lead to improvement in patient’s compliance, reduced cost for equipment cleaning, and prevention of unpleasant odors.

UVA irradiation alone showed a decrease in bacterial counts. Furthermore, the photocatalytic activities of TiO_2_ coating decreased significantly in bacterial counts of *S. gordonii*, *S. oralis* ATCC 35037 (Figure 1), *S. sobrinus*, *S. mutans*, *S. oralis* GTC, *S. sanguinis*, and *S. mitis* (Figure 2). When uncoated resins were irradiated with UVA light, all the bacteria reduced in counts. These reductions in the light(+)coat(−) groups were expected outcomes and followed a similar trend as a previous study, which reported that the viability of *S. mutans* decreased significantly after 60 min of UVA irradiation as compared to the control which was not irradiated [43]. The decrease may be associated with the cell-damaging effect of UVA. Coat(+)light(+) group showed a higher antibacterial effect, as compared to the coat(−)light(+) group. The difference of antibacterial effect between these group was explained in the Table 1.

The primary step in photocatalytic decomposition consists of hydroxyl radical attack on the bacterial cell wall [48]. This leads to increased permeability which allows radicals to reach and damage the cytoplasmic membrane causing lipid peroxidation and thereby causing membrane disorder [48]. The antibacterial effect of TiO_2_ is associated to this disorder of cytoplasmic membrane [48,56]. We propose that the observed bacterial type-dependent variation in the antimicrobial effects may be due to differential effects of hydroxyl radicals on distinct bacterium species [57,58]. The antibacterial effect of the TiO_2_ coating for various organisms is determined primarily by the complexity and density of the cell walls, as well as by the types of micro-organisms [59].

In this study, we observed that UVA irradiation has antibacterial effect. In cariogenic bacteria, *S. mutans* was more resistant to UVA than *S. sobrinus*. This may be because of the higher GC content of *S. sobrinus* than *S. mutans*. It has been proposed that species with genomes exhibiting a high GC content are more susceptible to UV-induced mutagenesis [60]. Also, our results showed that the coefficients for *S. sanguinis* and *S. gordonii* in light(+) treatment were low. Consistently, it has been demonstrated that *S. sanguinis* and *S. gordonii* have higher resistance to H_2_O_2_ [61].

In all bacteria, *S. mutans* was the most resistant to UVA. Conversely, *S. sobrinus* and *S. oralis* were highly susceptible to UVA. Several factors may contribute to the cause of these variable responses to UVA among species. For instance, the production of various ROSs involved in inducing UV damage may vary among species. In addition, the method of defending and repairing DNA damage differs among bacteria [61,62,63]. Also, the oxidative damage to biomolecules and counteracting protective mechanisms underlie the variability in UVA sensitivity among different bacterial species [64]. However, the reason of higher sensitivity of *S. oralis* and *S. sobrinus* to UV irradiation is unknown (Table 1). Further experiments are needed to understand the mechanistic basis for the variable susceptibility of oral bacteria to UVA.

In general, orthodontic patients are young, and oral care is a major problem for these patients. *S. mutans* and *S. sobrinus* are the most harmful cariogenic bacteria and TiO_2_ coating have shown to be effective against them.

Bacteria form biofilms on the surface of the device. During biofilm formation, adherent bacteria produce a polymer matrix in which the community becomes embedded and biofilm bacteria are notoriously resistant to antimicrobial substances [65]. Once they are established on the exposed surface of a dental device, removal of biofilms can be extremely difficult. Effective methodology for cleaning of dental device is not well established. Application of photocatalyst is considered one of the potential strategy to overcome these problems. Therefore, further study is necessary for understanding the effects of TiO_2_ on various organisms not only in vitro conditions but also in vivo and intraoral conditions.

With regard to flexural strength, there was no significant difference in bending strength even after irradiation for a long time. UV irradiated specimens fulfilled the requirements of the ISO 20795-2 standard for Fs testing (Figure 4A). Similarly, regarding strength modulus, there was no difference in bending strength even after irradiation for a long time (Figure 4B). These data imply that the resin can withstand irradiation for a long time to ensure a long-term clinical use of orthodontics. Even when irradiating for about 2 years, which is the recommended use period of the retainer, the durability was satisfactory. It was shown that clinical application is achievable.

Moreover, TiO_2_-coating improved the hydrophilic properties of the surface of the denture base acrylic resin (Figure 5A,B). A previous study reported that the water contact angle of surfaces in TiO_2_-coated resin was 68.1 ± 3.4 degrees [66]. However, TiO_2_-coating makes the resin surface more hydrophilic, with a water contact angle of 25.4 ± 2.1 degrees (Figure 5). It has been reported that TiO_2_ coating applied to acrylic resin inhibits the adhesion of *S. sanguinis* and *C. albicans* organisms [67,68]. In this study, enhancement in the hydrophilic properties of acrylic resin based orthodontic resin surface suppresses the adhesion of early colonizer, the subsequent adhesion of other microbes, which could reduce the total number of microbes adhering to orthodontic resin. Suppression of early colonizer could reduce further bacterial adhesion thereby reducing the risk of systemic disease. Moreover, improvement in the hydrophilic properties of orthodontic resin surface can suppress adhesion of other dirt such as food debris. Even without irradiation, the coat(+) group showed higher hydrophilicity making it easier to remove dirt.

We would like to establish the novel home care method for orthodontic retainer, with use of TiO_2_-coating and UV irradiation. As one of the clinical applications, patients place the retainer under the UV lamp and they can also easily clean it at home. This cleaning methods is very simple. In addition, cleaning up the device can be carried out by a professional at the time of visit to the clinic. Our method can be applied not only to a retainer but also to other orthodontic appliances (expansion plate, functional orthodontic appliance), as well as to denture base and occlusal splint.

One of the important aspect of our method is biocompatibility. It has been reported that in animals the TiO_2_-coated resin has no irritation to the oral mucosa, nor does it cause skin sensitization. Any elution of components from the coating has no deleterious effects on the tissues [69].

Overall, we demonstrate that the TiO_2_-coated resin exposed to UVA irradiation shows great reduction of microbial counts when compared with uncoated and coated without UVA-exposed samples. In addition, the durability of the specimen showed a higher value than the required standard value, indicating that the effect of irradiation was small. In conclusion, the results of this preliminary study suggest that the antibacterial effect of TiO_2_-coated resin can be beneficial in long-lasting orthodontic treatments.

## 5. Conclusions

The antimicrobial activity of the resin coated with TiO_2_ was examined by bacterial count of the early colonizers and cariogenic bacteria under UV irradiation. The results of present study suggest that coating with the ultraviolet responsive photocatalyst TiO_2_ is useful for antimicrobial properties of removable orthodontic resin based retainer.

## Figures and Tables

**Table 1:** Models of three way ANOVA for changes in bacterial counts. *p*-values less than 0.05 were considered statistically significant.

		***S. gordonii*** **ATCC10558**			***S. mitis***		***S. oralis*** **ATCC35037**				***S. oralis GTC276***		
		**Coefficient**	**95% CI**	***p*-Value**	**Coefficient**	**95% CI**	***p*-Value**	**Coefficient**	**95% CI**	***p*-Value**	**Coefficient**	**95% CI**	***p*-Value**
Intercept		6.563	6.04–7.08	0.999<	8.267	7.57–8.96	0.999<	6.584	6.18–6.98	0.999<	8.840	8.30–9.37	0.999<
time	30 min	−0.061	−0.67–0.55	0.845	−0.306	−1.13–0.52	0.462	−0.372	−0.84–0.10	0.123	−0.498	−1.13–0.13	0.122
	60 min	−0.238	−0.85–0.37	0.444	−0.753	−1.57–0.07	0.073	−0.616	−1.09–−0.14	0.012	−0.700	−1.33–−0.06	0.031
	90 min	−0.458	−1.07–0.15	0.143	−1.246	−2.07–−0.42	<0.001	−0.846	−1.32–−0.37	<0.001	−0.877	−1.51–−0.24	<0.001
	120 min	−1.001	−1.61–−0.38	<0.001	−1.671	−2.49–−0.84	<0.001	−1.003	−1.47–−0.52	<0.001	−1.260	−1.89–−0.62	<0.001
	150 min	−1.175	−1.79–−0.55	<0.001	−1.935	−2.75–−1.10	<0.001	−1.231	−1.70–−0.75	<0.001	−1.576	−2.21–−0.94	<0.001
	180 min	−1.654	−2.27–−1.03	<0.001	−2.432	−3.25–−1.60	<0.001	−1.491	−1.96–−1.01	<0.001	−1.820	−2.45–−1,18	<0.001
coat(+)		−0.001	−0.46–0.46	0.996	−0.015	−0.63–0.60	0.963	0.017	−0.34–0.37	0.925	−0.008	−0.48–0.47	0.975
light(+)		−0.695	−1.16–−0.22	<0.001	−1.457	−2.08–−0.83	<0.001	−1.247	−1.60–−0.88	<0.001	−1.338	−1.81–−0.85	<0.001
interaction of light and coat		−1.216	−1.87–−0.55	<0.001	−1.845	−2.72–−0.96	<0.001	−0.727	−1.23–−0.21	0.006	−1.206	−1.88–−0.52	<0.001
		***S. mutans***			***S. sobrinus***			***S. sanguinis***					
		**Coefficient**	**95% CI**	***p*-Value**	**Coefficient**	**95% CI**	***p*-Value**	**Coefficient**	**95% CI**	***p*-Value**			
Intercept		5.609	5.38–5.83	0.999<	11.066	10.68–11.44	0.999<	9.280	8.90–9.65	0.999<			
time	30 min	−0.119	−0.38–0.14	0.376	−0.585	−1.03–−0.13	0.011	−0.105	−0.54–0.33	0.638			
	60 min	−0.272	−0.53–−0.01	0.044	−0.938	−1.38–−0.49	<0.001	−0.231	−0.67–0.20	0.299			
	90 min	−0.347	−0.61–−0.82	0.011	−1.121	−1.56–−0.67	<0.001	−0.348	−0.78–0.09	0.120			
	120 min	−0.512	−0.77–−0.24	<0.001	−1.146	−1.59–−0.70	<0.001	−0.718	−1.15–−0.27	0.002			
	150 min	−0.512	−0.77–−0.24	<0.001	−1.404	−1.85–−0.95	<0.001	−0.848	−1.28–−0.40	<0.001			
	180 min	−0.908	−1.17–−0.64	<0.001	−1.555	−2.00–−1.10	<0.001	−1.239	−1.67–−0.79	<0.001			
coat(+)		<0.001	−0.20–0.20	0.998	0.001	−0.33–0.33	0.995	<0.001	−0.33–0.33	0.999<			
light(+)		−0.480	−0.67–−0.27	<0.001	−1.587	−1.92–−1.25	<0.001	−0.514	−0.84–−0.18	0.003			
interaction of light and coat		−0.557	−0.84–−0.27	<0.001	−0.680	−1.15–−0.20	0.006	−0.948	−1.41–−0.47	<0.001			
